# Role of dietary amino acid balance in diet restriction‐mediated lifespan extension, renoprotection, and muscle weakness in aged mice

**DOI:** 10.1111/acel.12796

**Published:** 2018-06-25

**Authors:** Shohei Yoshida, Kosuke Yamahara, Shinji Kume, Daisuke Koya, Mako Yasuda‐Yamahara, Naoko Takeda, Norihisa Osawa, Masami Chin‐Kanasaki, Yusuke Adachi, Kenji Nagao, Hiroshi Maegawa, Shin‐ichi Araki

**Affiliations:** ^1^ Department of Medicine Shiga University of Medical Science Otsu Shiga Japan; ^2^ Department of Medicine IV Faculty of Medicine University of Freiburg Freiburg Germany; ^3^ Department of Diabetology and Endocrinology Kanazawa Medical University Kahoku‐Gun Ishikawa Japan; ^4^ Frontier Research Labs Institute for Innovation Ajinomoto Co., Inc. Kawasaki Kanagawa Japan

**Keywords:** amino acids, diet restriction, H_2_S, kidney aging, methionine, trans‐sulfuration pathway

## Abstract

Extending healthy lifespan is an emerging issue in an aging society. This study was designed to identify a dietary method of extending lifespan, promoting renoprotection, and preventing muscle weakness in aged mice, with a focus on the importance of the balance between dietary essential (EAAs) and nonessential amino acids (NEAAs) on the dietary restriction (DR)‐induced antiaging effect. Groups of aged mice were fed ad libitum, a simple DR, or a DR with recovering NEAAs or EAAs. Simple DR significantly extended lifespan and ameliorated age‐related kidney injury; however, the beneficial effects of DR were canceled by recovering dietary EAA but not NEAA. Simple DR prevented the age‐dependent decrease in slow‐twitch muscle fiber function but reduced absolute fast‐twitch muscle fiber function. DR‐induced fast‐twitch muscle fiber dysfunction was improved by recovering either dietary NEAAs or EAAs. In the ad libitum‐fed and the DR plus EAA groups, the renal content of methionine, an EAA, was significantly higher, accompanied by lower renal production of hydrogen sulfide (H_2_S), an endogenous antioxidant. Finally, removal of methionine from the dietary EAA supplement diminished the adverse effects of dietary EAA on lifespan and kidney injury in the diet‐restricted aged mice, which were accompanied by a recovery in H_2_S production capacity and lower oxidative stress. These data imply that a dietary approach could combat kidney aging and prolong lifespan, while preventing muscle weakness, and suggest that renal methionine metabolism and the trans‐sulfuration pathway could be therapeutic targets for preventing kidney aging and subsequently promoting healthy aging.

## 1 INTRODUCTION

The normal aging process involves a deterioration in the function of many organs, including that of the kidney. The high prevalence of chronic kidney disease (CKD) in the elderly population is recognized as a major health problem (Tonelli & Riella, 2014), because it is associated with higher risks of mortality, cardiovascular events, muscle wasting, and cognitive impairment (Go, Chertow, Fan, McCulloch, & Hsu, 2004; Kurella et al., 2005; Workeneh & Mitch, 2010). Therefore, a strategy to combat kidney aging is now urgently required.

Dietary restriction (DR) can extend lifespan in numerous species, including mammals, and prevent age‐related impairment in organ function, including kidney dysfunction (Colman et al., 2014; Kume et al., 2010; Lin, Ford, Haigis, Liszt, & Guarente, 2004; Shimokawa et al., 2015). In contrast, DR has only one principal negative effect: muscle weakness due to protein wasting (Lopes, Russell, Whitwell, & Jeejeebhoy, 1982; Thomas, 2007), which also represents a health problem in both elderly subjects and patients with kidney disease (Goodpaster et al., 2006; Workeneh & Mitch, 2010). Recently, a beneficial effect of dietary protein or amino acid supplementation on muscle weakness has been proposed (Paddon‐Jones & Rasmussen, 2009; Paddon‐Jones, Short, Campbell, Volpi, & Wolfe, 2008; Volpi, Kobayashi, Sheffield‐Moore, Mittendorfer, & Wolfe, 2003). However, there has been concern that such a supplement may adversely affect the progression of CKD and reduce DR‐induced health benefits. Thus, a dietary regimen that could maintain a better quality of life in the aging population has not been identified to date.

Amino acids are classified into two groups: essential (EAAs) and nonessential amino acids (NEAAs) (Supporting Information Table S1). It is well known that each amino acid has a different biological effect. Of particular note is that, in a study conducted in *Drosophila*, recovering dietary EAA, but not NEAA, prevented DR‐induced lifespan extension (Grandison, Piper, & Partridge, 2009). Given that the beneficial effect of simple DR on lifespan is observed in most living organisms (Colman et al., 2014; Kume et al., 2010; Lin et al., 2004), the distinct effects of dietary EAA and NEAA on DR‐mediated longevity may be conserved across species. We therefore hypothesized that the balance between the dietary content of EAAs and NEAAs would affect DR‐induced lifespan extension and modify age‐related organ dysfunction in mammals, with implications for the design of an improved dietary regimen aimed at extending healthy lifespan.

To test this hypothesis, we examined the effect of recovering either dietary EAAs or NEAAs on DR‐induced lifespan elongation, renoprotection, and muscle weakness in aged mice. Our results demonstrate that a diet containing mainly EAAs prevents the health benefits of DR and that, among the nine EAAs, intrarenal methionine metabolism and the trans‐sulfuration pathway regulate kidney aging and longevity in mice.

## 2 RESULTS

### 2.1 Role of dietary amino acid balance in DR‐induced lifespan extension in mice

**Figure 1:**
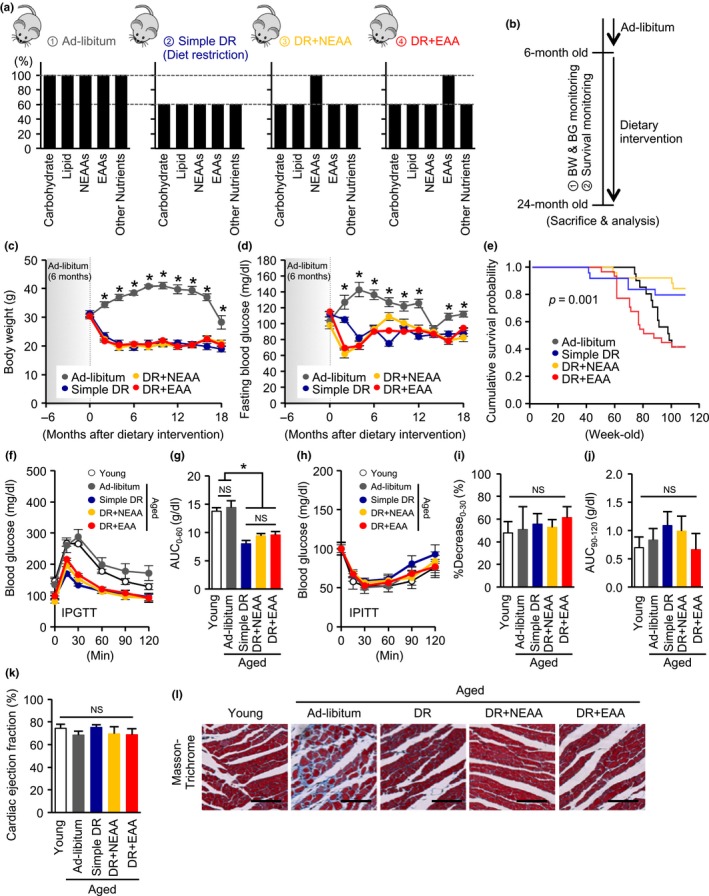
Effect of recovering dietary amino acids on dietary restriction‐induced lifespan prolongation and health benefits in mice. (a) Dietary regimens in four experimental groups of aged mice: ad libitum, simple diet restriction (DR), DR with recovering all nonessential amino acids (NEAA), and DR with recovering all essential amino acids (EAA). (b) Time schedule in the dietary intervention study. Dietary intervention was started at 6 months of age and continued until 24 months. During the follow‐up period, body weight (BW), blood glucose (BG), and survival rate were monitored. (c, d, e) BW (c), fasting BG (d), and cumulative survival probability (e) during the study. (f, g) Blood glucose (f) and area under the curve (AUC) (g) during an intraperitoneal glucose tolerance test (IPGTT) in the five mouse groups: young, ad libitum fed aged, simple diet restriction (DR), DR with recovering all nonessential amino acids (NEAA), and DR with recovering all essential amino acids (EAA). (h, i) Blood glucose (h) and reduction rate of glucose level in the first 30 min (i), the AUC of glucose levels in the last 30 min (j) during an intraperitoneal insulin tolerance test (IPITT). (k) Cardiac ejection fraction, determined using echocardiography. (l) Representative photomicrographs of sections stained with Masson's trichrome prepared from cardiac samples taken from the five groups of mice. Error bar = 100 μm. All data are expressed as mean ± *SEM*. *p < 0.01 vs. the indicated group. NS indicates no statistically significance

We first established four dietary intervention groups of aged mice (Figure 1a). The detailed composition of the diets is shown in Supporting Information Table S2. The dietary intervention was started at 6 months of age and continued until 24 months (Figure 1b). The DR‐induced reductions in body weight (BW) and blood glucose level were not influenced by the supplement of any dietary amino acids (Figure 1c,d). Consistent with previous reports (Forster, Morris, & Sohal, 2003; Mitchell et al., 2016), introduction of 40% DR tended to increase mortality in the first 20% of the survival curve but ultimately extended lifespan, when compared with the ad libitum‐fed aged mouse group (Figure 1e). Although the replenishment of NEAA did not affect the DR‐mediated lifespan extension, it is significant that the addition of EAA not only abrogated the beneficial effects of DR on lifespan, but also seemed to have detrimental effects (Figure 1e).

### 2.2 Effects of DR and dietary amino acids on organs function in aged mice

Although simple DR reduced total plasma protein levels in aged mice, no significant differences in any other general laboratory data were observed among the groups of aged mice (Supporting Information Table S3). Cancer development, which is associated with higher serum IGF‐1 levels, is a cause of death in aged mice (Rincon, Rudin, & Barzilai, 2005). Simple DR reduced both serum IGF‐1 and tumor prevalence at necropsy in dead mice, and these effects were not altered by the replenishment of the diet with either NEAA or EAA (Supporting Information Tables S3 and S4).

Simple DR led to better glucose tolerance, determined using an intraperitoneal glucose tolerance test (IPGTT), in aged mice, and this was not affected by recovering either NEAAs or EAAs (Figure 1f,g). Both the rate of reduction in blood glucose in the first 30 min and the area under the curve in the final 30 min during an intraperitoneal insulin tolerance test (IPITT) did not differ among the groups of mice (Figure 1h–j), suggesting that the replenishment of the diet with either NEAA or EAA did not alter insulin resistance and gluconeogenesis in the DR aged mice.

Cardiac ejection fraction was not different among the groups of aged mice (Figure 1k). Although fibrotic lesion, identified by Masson's trichrome staining, was clearly apparent in the hearts of the ad libitum‐fed aged mice, whereas it was not evident in the hearts of the DR plus EAA group, which had higher mortality (Figure 1l).

### 2.3 Effects of DR and dietary amino acids on muscle phenotypes in aged mice

**Figure 2:**
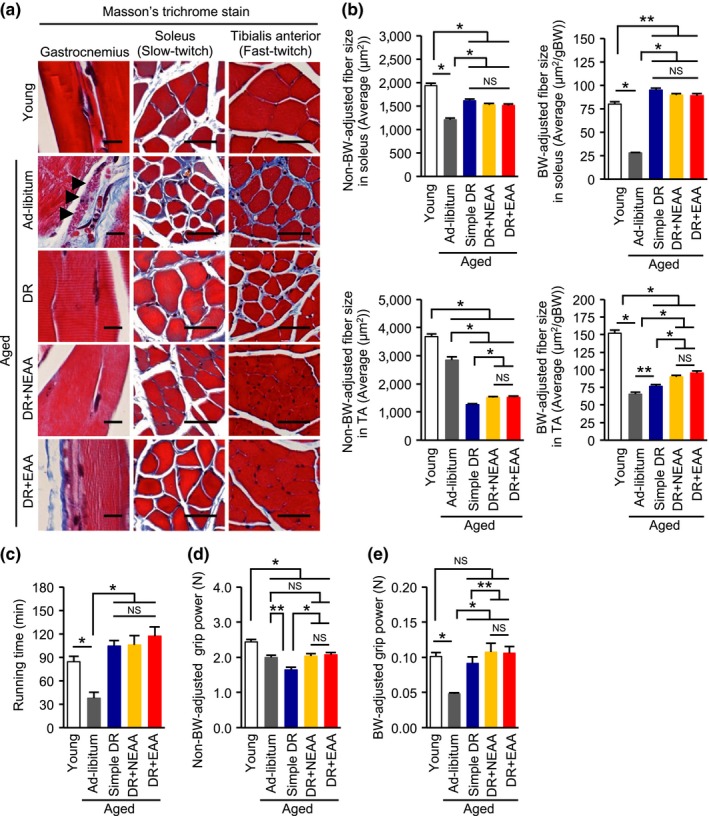
Effect of replenishing dietary amino acids on age‐related and dietary restriction‐related muscle weakness. (a) Representative photomicrographs of sections stained with Masson's trichrome of gastrocnemius (GAS), soleus, and tibialis anterior (TA) muscles from the five groups of mice. Scale bars = 50 μm. (b) Nonbody weight (BW)‐adjusted and BW‐adjusted muscle fiber sizes in the soleus and TA. (c) Maximal running time in a treadmill study. (d, e) Non‐BW‐adjusted absolute grip power (d) and BW‐adjusted grip power (e). All data are expressed as mean ± *SEM*. *p < 0.01 vs. the indicated group. **p < 0.05 vs. the indicated group. NS indicates no statistically significance

Age‐dependent lipofuscin deposition in the gastrocnemius samples was observed only in the group of ad libitum‐fed aged mice (Figure 2a). Fibrosis was observed in the soleus, a muscle rich in slow‐twitch muscle fibers, of the ad libitum‐fed aged mice (Figure 2a). Furthermore, both non‐BW‐adjusted and BW‐adjusted muscle fibers in the soleus of the ad libitum‐fed aged mice were significantly smaller than those in the other groups (Figure 2b; Supporting Information Figure S1A). Consistent with the results of the histological analysis, endurance running capacity, reflecting the function of slow‐twitch muscle fibers, was lower in the aged mice (Figure 2c). The age‐dependent histological and functional alterations in slow‐twitch muscle fibers were significantly ameliorated by DR, regardless of whether amino acids were replenished.

Both non‐BW‐adjusted and BW‐adjusted grip power decreased with age, indicative of poorer fast‐twitch muscle fiber function (Figure 2d,e). Although simple DR further reduced non‐BW‐adjusted absolute grip power (Figure 2d), it improved BW‐adjusted grip power (Figure 2e). However, regardless of whether absolute or BW‐adjusted figures for grip power were considered, both NEAA and EAA supplementation increased grip power in the DR mice (Figure 2d,e). These functional changes were consistent with the histological findings of fibrosis and altered fast‐twitch muscle fiber size in the tibialis anterior (Figure 2b; Supporting Information Figure S1B).

### 2.4 Effects of DR and dietary amino acids on glomerular injury in aged mice

**Figure 3:**
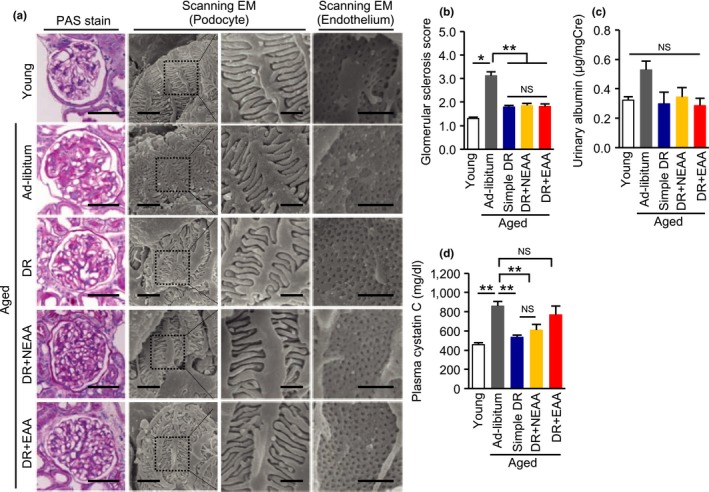
Effect of recovering dietary amino acids on the diet restriction‐induced amelioration of glomerular damage in aged mice. (a) Representative pictures of PAS staining, and scanning electron microscopy (EM) of podocytes and glomerular endothelium in the five mouse groups: young, ad libitum fed aged, simple diet restriction (DR), DR with recovering all nonessential amino acids (NEAA), and DR with recovering all essential amino acids (EAA). Scale bars = 50 μm (PAS), 3 and 2 μm (left and right lanes of podocytes), 1 μm (endothelium). (b) Semi‐quantitative data for PAS‐positive glomerular sclerotic lesions. (c) Urinary albumin excretion levels. (d) Plasma cystatin C levels. All data are expressed as mean ± *SEM*. *p < 0.01 vs. the indicated group. **p < 0.05 vs. the indicated group. NS indicates no statistical significance

The PAS‐positive area in the glomerular region was larger in aged mice than in young mice (Figure 3a,b), which was accompanied by a trend toward an increase in albuminuria (Figure 3c). Although alterations to podocyte foot processes were not observed, endothelial cell damage, characterized by disappearance of fenestrae under scanning electron microscopy, was observed in aged mice, suggesting that endothelial cell dysfunction is likely responsible for the albuminuria observed in aged mice (Figure 3a). Glomerular sclerotic change, the disappearance of fenestrae, and albuminuria were all ameliorated by DR, irrespective of the nature of the amino acid supplementation (Figure 3a–c).

### 2.5 Effects of DR and dietary amino acids on renal tubulointerstitial pathology in aged mice

**Figure 4:**
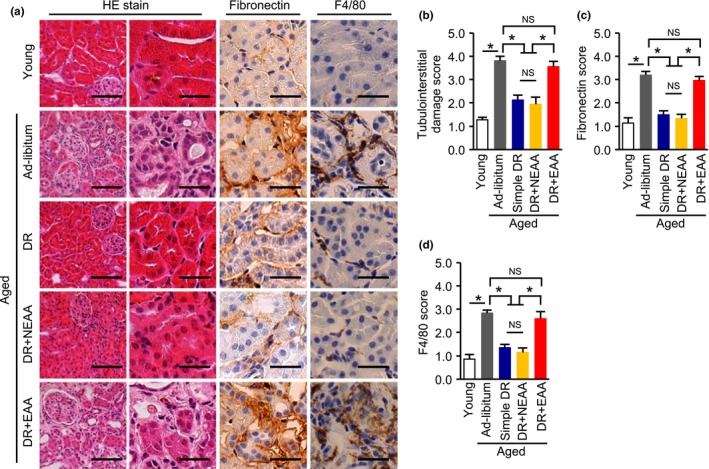
Effect of recovering dietary amino acids on the dietary restriction‐induced amelioration of tubulointerstitial lesions in aged mice. (a) Representative photomicrographs of sections stained with HE, and immunostaining for fibronectin and F4/80‐positive macrophage, in the five mouse groups: young, ad libitum fed aged, simple diet restriction (DR), DR with recovering all nonessential amino acids (NEAA), and DR with recovering all essential amino acids (EAA). Scale bars = 100 μm (Left in HE) and 30 μm (other lanes). (b, c, d) Semi‐quantitative data representing tubular cell damage (b), fibronectin deposition (c), and F4/80‐positive macrophage infiltration (d) in the five groups. All data are expressed as mean ± *SEM*. *p < 0.01 vs. the indicated group. NS indicates no statistical significance

No significant differences in glomerular phenotype were observed among the aged groups of mice. However, the DR‐induced reduction in plasma cystatin C in aged mice was canceled by recovering dietary EAAs but not NEAAs (Figure 3d). We then evaluated tubulointerstitial lesions, which are more responsible for impairments in renal function than are glomerular lesions, as part of any kidney disease. Consistent with the plasma cystatin C data, DR‐induced amelioration of tubular cell damage, fibronectin deposition, and F4/80‐positive macrophage infiltration were re‐worsened by recovering dietary EAAs but not NEAAs (Figure 4a–d).

### 2.6 Renal phenotype at necropsy

Of the organs examined in the mice that survived, kidney pathology in particular tended to be most evident in groups with a higher mortality rate. We therefore thought that kidney injury might be responsible for the higher mortality rate in the EAA group and analyzed the renal phenotype at necropsy in mice that died (Supporting Information Figure S2). Renal fibrosis was not clearly apparent in the kidneys of dead mice in the simple DR and DR plus NEAA groups, and was severe in older mice of the ad libitum‐fed group. In contrast, renal fibrosis was very severe, even in mice that died in middle age, in the DR plus EAA group.

### 2.7 Effects of DR and dietary amino acids on intrarenal amino acid profile in aged mice

**Figure 5:**
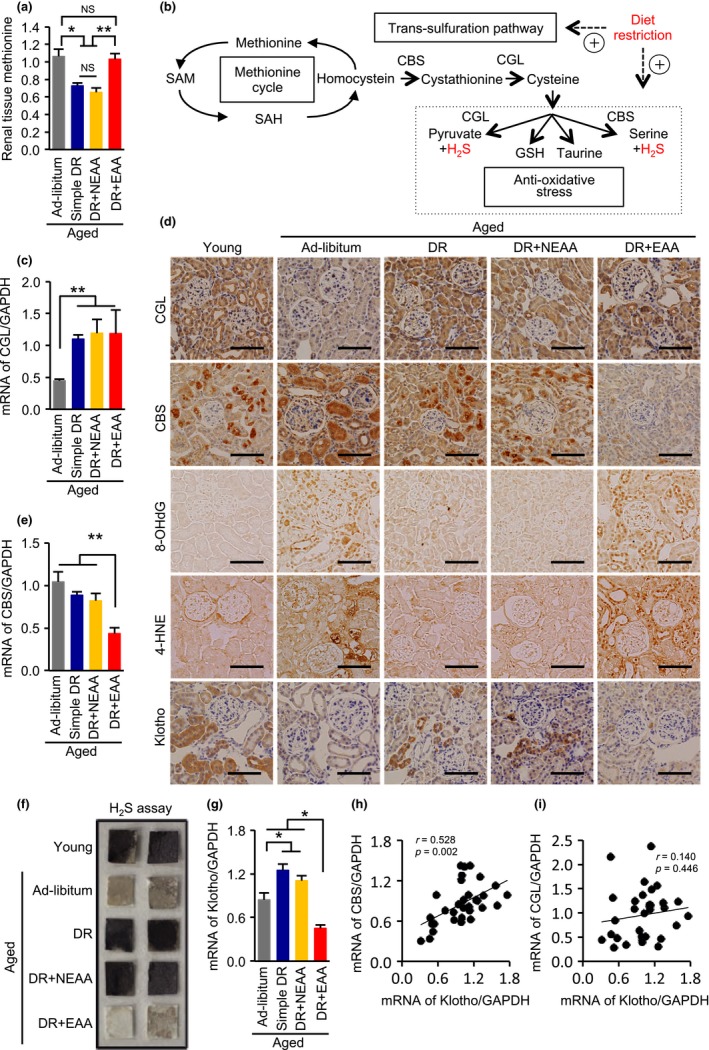
Effects of dietary restriction and dietary amino acids on methionine levels and the trans‐sulfuration pathway in aged kidney. (a) Renal methionine content in the four groups of aged mice: ad libitum aged, simple diet restriction (DR), DR with recovering all nonessential amino acids (NEAA), and DR with recovering all essential amino acids (EAA). (b) Schema of methionine metabolism and the trans‐sulfuration pathway. H_2_S activates antioxidative stress mechanisms. (c) Renal mRNA expression levels of CGL. GAPDH was used as an internal control. (d) Representative photomicrographs of sections immunostained for CGL, CBS, 8‐OHdG, 4‐hydroxynoneal (4‐HNE) or klotho in the five mouse groups. Scale bars = 100 μm. (e) Renal mRNA expression levels of CBS. GAPDH was used as an internal control. (f) H_2_S production capacity in kidney homogenates, determined using lead acetate H_2_S detection paper. Darkening of the lead paper indicates high H_2_S production capacity. (g) mRNA expression level of klotho. GAPDH was used as an internal control. (H, I) Correlation analysis of CBS, CGL, and klotho mRNA expression. All data are expressed as mean ± *SEM*. *p < 0.01 vs. the indicated group. **p < 0.05 vs. the indicated group. NS indicates no statistical significance

Whether there is some specificity to the effects of restriction of different essential amino acids is still under debate (Longo et al., 2015). Therefore, the amino acid profiles of homogenates of kidney samples obtained from the four groups of aged mice were evaluated, to identify an amino acid that might be responsible for the adverse effects of EEA supplementation in the diet‐restricted aged mice. Of the assayed amino acids, methionine levels were significantly higher in the kidneys of the ad libitum*‐*fed and EAA groups of mice (Figure 5a; Supporting Information Figure S3), which had significantly more severe kidney pathology than the other groups.

Because the trans‐sulfuration pathway, which is a branch of the methionine cycle and produces hydrogen sulfate (H_2_S), has recently been reported to be involved in the antioxidant effect of short‐term DR in mammalian tissues (Hine et al., 2015) (Figure 5b), we measured the activity of this pathway. DR increased mRNA and protein expression levels of a key enzyme in the trans‐sulfuration pathway, cystathionine γ‐lyase (CGL), regardless of the nature of the dietary amino acid supplementation (Figure 5c,d). In contrast, recovering dietary EAAs decreased expression levels of mRNA and protein of another key enzyme in the pathway, cystathionine β‐synthase (CBS) (Figure 5d,e). H_2_S production capacity, determined using the lead sulfate paper method (Hine et al., 2015)_,_ was lower in the kidney of aged mice than in young mice (Figure 5f). Furthermore, consistent with the change in CBS expression, the DR‐induced increase in H_2_S production capacity was inhibited by recovering EAAs in the diet (Figure 5f). Accordingly, the DR‐induced amelioration of the age‐dependent increase in renal oxidative stress, determined by 8‐hydroxy‐2′‐deoxyguanosine (8‐OHdG) and 4‐hydroxy‐2‐nonena (4‐HNE) staining, was canceled by EAA supplementation (Figure 5d).

### 2.8 A possible link between klotho and CBS expression levels in aged mice

The mRNA and protein expression levels of the antiaging and renoprotective gene, klotho, which encodes a protein that is secreted from the kidney, were lower in the kidney of the ad libitum and the DR plus EAAs aged mice than those in the simple DR and the DR plus NEAAs aged mice (Figure 5d,g), consistent with the severity of the kidney pathology and mortality rates. To investigate whether CBS or CGL is more associated with the antiaging molecule, klotho, we analyzed the correlations between klotho levels and CBS and CGL levels in the kidney samples. Renal klotho expression levels showed a significant positive correlation with the CBS expression levels, but not the CGL expression levels, in aged kidneys (Figure 5h,i), suggesting that renal CBS, rather than CGL, might play a more important role in the effect of DR to ameliorate renal senescence.

### 2.9 Effect of methionine removal on EAA supplementation‐induced adverse effects in diet‐restricted aged mice

To determine whether methionine is involved in the mechanism of the adverse effects of EAA replacement in diet‐restricted aged mice, we established an additional mouse group, DR mice consuming a diet containing a normal level of EAAs, except for that of methionine, and compared its lifespan and renal phenotype with simple DR and DR plus EAAs groups.

**Figure 6:**
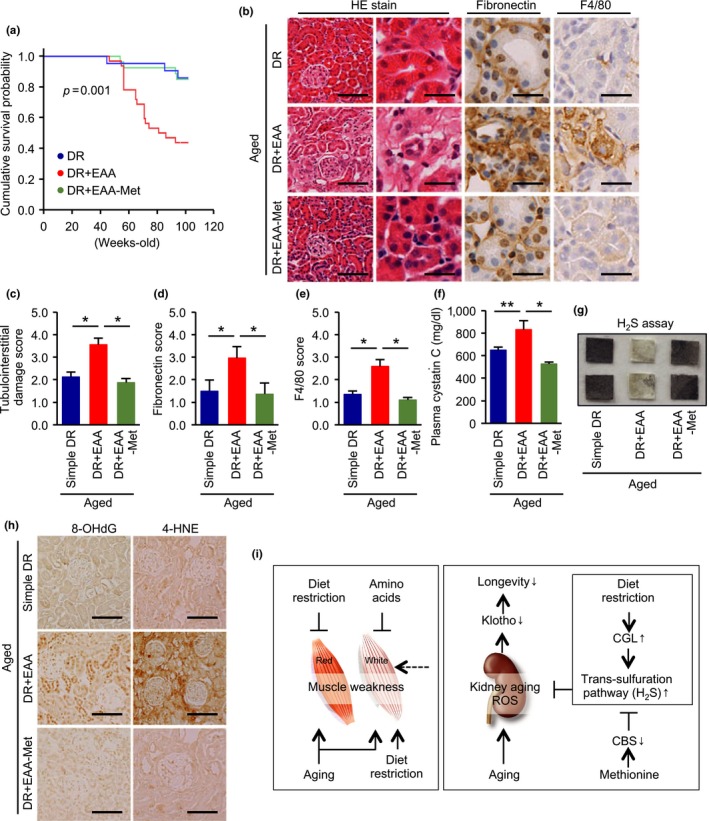
Effect of methionine removal from the dietary essential amino acid supplement on the diet restriction‐induced health benefits. (a) Cumulative survival probability in three groups of aged mice: simple diet restriction (DR), DR with recovering all essential amino acids (EAA), and DR with recovering all essential amino acids except methionine. (b) Representative photomicrographs of HE staining, and immunostaining for fibronectin and F4/80‐positive macrophages. Scale bars = 100 μm (left in HE) and 30 μm (other lanes). (c, d, e) Semi‐quantitative data representing tubular cell damage (c), fibronectin deposition (d), and F4/80‐positive macrophage infiltration (e). (f) Plasma cystatin C levels. (g) H_2_S production capacity in kidney homogenates, determined using lead acetate H_2_S detection paper. Darkening of the lead paper indicates high H_2_S production capacity. (h) Representative photomicrographs of immunostaining for 8‐OHDG and 4‐hydroxunoneal (4‐HNE) in kidney sections. Scale bars = 100 μm. (i) Proposed hypothesis. The normal aging process involves deterioration in function of both slow‐ and fast‐twitch fiber muscles. Simple diet restriction (DR) and dietary amino acid supplementation can ameliorate age‐dependent red fiber muscle weakness and DR‐induced fast‐twitch fiber muscle weakness, respectively. DR has a renoprotective effect during aging, which is prevented by dietary supplementation with methionine, an essential amino acid. Methionine inhibits the intrarenal trans‐sulfuration pathway and therefore decreases intrarenal H_2_S production, leading to oxidative stress in the kidney. All data are expressed as mean ± *SEM*. *p < 0.01 vs. the indicated group. **p < 0.05 vs. the indicated group. NS indicates no statistical significance

The adverse effect of recovering dietary EAAs on DR‐induced lifespan extension was prevented by the removal of methionine (Figure 6a). Furthermore, this dietary regimen ameliorated the EAA supplementation‐induced exacerbation of tubulointerstitial lesions and the increase in plasma cystatin C (Figure 6b–f). These effects were accompanied by greater H_2_S production capacity and a reduction in renal oxidative stress (Figure 6g,h).

## 3 DISCUSSION

This study has demonstrated the distinct effects of dietary EAAs and NEAAs on the DR‐induced increase in healthy lifespan in mice. Dietary supplementation with either EAAs or NEAAs was beneficial for the prevention of DR‐induced muscle weakness, the principal complication of DR (Figure 6i). Interestingly, however, recovering dietary EAAs, but not NEAAs, canceled the DR‐induced prolongation of lifespan and renoprotection. We have, for the first time, shown the importance of EAA/NEAA balance in DR‐induced health benefits in mammals, which has previously only been observed in the *Drosophila* study (Grandison et al., 2009). It appears that the distinct effects of dietary EAAs and NEAAs on DR‐induced longevity are conserved across species.

In this study, we have shown distinct effects of dietary EAAs and NEAAs during DR on mortality in 24‐month‐old (middle aged) mice. However, it is still unclear whether the effects of dietary manipulation persist in very old organisms. An observational study in humans has shown that low protein intake during middle age (50–65 years) is associated with major reductions in cancer prevalence and overall mortality, while higher protein intake is associated with the opposite outcomes in populations over 65 years (Levine et al., 2014). These results indicate that dietary regimens do not affect healthy lifespan in the same way throughout life, and therefore, the diet should be modified according to age to maximize healthy lifespan. Thus, a longer follow‐up study in mice is needed to investigate whether the distinct effects of dietary EAAs and NEAAs during DR can be observed even in very old mammals.

This study has shown that the balance between dietary EAAs and NEAAs affected age‐related kidney dysfunction. Recent observational studies have shown that a high intake of red meat increases the risk of end‐stage renal disease (ESRD), whereas intake of nuts decreases the prevalence of ESRD in the general population (Haring et al., 2017; Lew et al., 2017). Given that EAAs, especially methionine, are predominantly found in animal‐based proteins, rather than plant‐based proteins (Ables & Johnson, 2017), an imbalance between these protein sources in the diet might influence kidney function, and the substitution of alternative sources of protein may reduce the incidence of ESRD.

Whether there is a beneficial effect of DR on muscle function has been controversial to date, because whereas some studies have shown that DR improves muscle function (Marzetti, Lees, Wohlgemuth, & Leeuwenburgh, 2009; McKiernan et al., 2012), another showed it had the opposite effect (Lopes et al., 1982). Our results demonstrate that DR can improve slow‐twitch muscle fiber function, along with the maintenance of muscle fiber size. DR worsened fast‐twitch muscle fiber function when the analysis was conducted using the absolute value of grip power and fiber size, which might reflect enhanced muscle wasting due to DR. However, if the results were adjusted by body weight, DR did not decrease, more likely increased, grip power, suggesting that fast‐twitch muscle fiber function had improved. Thus, the apparent effect of DR on muscle function depends on the muscle type and the analytical method used, which might explain the inconsistencies in the results of previous DR studies. Furthermore, whether the maintenance of BW‐adjusted fiber size and power or an improvement in absolute muscle power are more important for a good quality of life in the elderly population remains to be determined in the future. However, it was apparent that both types of amino acid supplement ameliorated DR‐dependent muscle weakness in mice.

H_2_S is a product of the trans‐sulfuration pathway that has the potential to have physiological benefits, including the extension of lifespan (Cuevasanta, Denicola, Alvarez, & Moller, 2012; Zhang et al., 2013). Although H_2_S is very toxic at high concentrations, it is produced at low concentrations by the degradation of cysteine or homocysteine by CGL or CBS, and acts in the vasculature and the brain as a signaling molecule, reducing blood pressure (Yang et al., 2008), and preventing neurodegeneration (Paul & Snyder, 2012). Furthermore, exogenous H_2_S can also extend the lifespan of worms (Miller & Roth, 2007) and prevent kidney injury in some experimental models (Bos et al., 2009; Holwerda et al., 2014). Thus, it is apparent that H_2_S has a protective role in tissues in a number of species. Although neither the dietary requirement for H_2_S, nor the potential role of H_2_S in the benefits of DR, is well understood, a recent paper showed that greater H_2_S production via the trans‐sulfuration pathway is an evolutionarily conserved response to DR, with the potential to mediate a number of the benefits of DR, and that this effect was blunted by dietary supplementation with sulfur‐containing amino acids (methionine and cysteine) in mice (Hine et al., 2015). Furthermore, among several proposed mechanisms for the protective effect of H_2_S in tissues (Hine & Mitchell, 2015), a reduction in oxidative stress is most likely to play a central role. Our data provide an additional insight into the beneficial effect of the trans‐sulfuration pathway for health (Figure 6i).

The precise cause of the higher mortality in the DR plus EAAs aged mouse group is uncertain. In this study, serum IGF‐1 levels and tumor prevalence were significantly higher only in the ad libitum‐fed aged mice. These results are consistent with the well‐described connection between cancer development, high IGF‐1 levels, and death in aged mice, and suggest that the higher mortality in the DR plus EAA aged mice cannot be explained by high IGF‐1 or cancer. An effect of dietary EAA on the kidney may account for this discrepancy, because the effect on lifespan was associated with an altered kidney phenotype but not with effects in any other organ, such as heart or skeletal muscle, in the mice that survived. Specifically, very severe kidney fibrosis was observed in mice in the DR plus EAA group at necropsy. Thus, kidney damage is likely to be one of the reasons for the higher mortality in the DR plus EAA group. However, we cannot conclude that kidney injury alone account for the higher mortality in the DR plus EAA group, because we did not evaluate any other organs in detail at necropsy.

Klotho is a hormone secreted from the kidney (Kuro‐o et al., 1997), and low klotho levels are associated with progressive aging (Kurosu et al., 2005). In our study, mRNA expression CBS was strongly associated with the mRNA expression level of klotho in the aged kidney. Although a detailed molecular link between CBS and klotho has not been identified, downregulation of CBS by methionine supplementation might lead to severe renal injury through greater oxidative stress, resulting in lower klotho expression. Thus, klotho may represent a possible link among dietary EAA (methionine), kidney injury, and mortality (Figure 6i).

There were a few limitations to this study, and some additional issues remain to be addressed. First, a survival bias might affect our conclusions, because we only studied mice that had survived until the end of the experimental period. Second, the molecular mechanism underpinning the dietary EAA‐induced decrease in CBS is still unclear, although this was likely to be involved in the EAA‐induced suppression of the trans‐sulfuration pathway. Several mechanisms have been proposed that may underpin the DR‐induced increase in CGL expression, but the regulation of CBS expression has not been described to date. When these remaining issues have been addressed, additional novel insight will be provided into kidney aging and the role of amino acid metabolism.

Despite strong evidence of the benefits of DR in humans, difficulties with compliance prevent its widespread clinical applications. Here, we show that dietary supplementation with amino acids other than methionine did not alter the beneficial effects of DR and likely ameliorated the principal adverse effect of DR, muscle weakness. Thus, such supplementation may improve compliance with DR and represent an improvement in the dietary regimen necessary for prolongation of healthy lifespan and protection of the kidney against aging. We have also shown that the beneficial role of methionine removal in the health benefits originating from DR can be observed in a number of species, including rodents. Hopefully, this effect will also extend to higher primates, including humans.

## 4 EXPERIMENTAL PROCEDURES

### 4.1 Study approvals

All animal procedures were performed in accordance with the guidelines of the Research Center for Animal Life Science of Shiga University of Medical Science (SUMS) and were approved by the SUMS Animal Care and Use Committee (2014‐6‐8H).

### 4.2 Animal models

Eight‐week‐old male C57BL/6 mice were obtained from Clea Japan Inc. (Tokyo, Japan). The mice were fed a standard diet until 6 months of age. For study 1, four groups of aged mice were established: ad libitum fed (AL) (*n* = 20), simple DR (*n* = 25), DR plus EAAs (*n* = 30), and DR plus NEAAs (*n* = 24). Three‐month‐old mice were used as young control group (*n* = 15). For study 2, a DR group that was supplemented with EAAs, without methionine (*n* = 20), was also established, and its phenotype was compared with those of simple DR (*n* = 25) and DR plus EAAs (*n* = 30) groups. Based on previous reports (Bluher, Kahn, & Kahn, 2003; Forster et al., 2003; Migliaccio et al., 1999; Mitchell et al., 2016; Weindruch & Walford, 1982), the survival rate of male C57BL/6 mice is approximately 50%–60% at 24 months old. Thus, we decided to use 24 months as the end‐point of this study, which enabled us to analyze the effect of each dietary regimen on survival, cardiac function, glucose metabolism, muscle function, histology, and serum parameters in living middle aged and older mice, but not in very old mice. The amount of food given to DR groups was reduced by 40% of the dietary amount of the ad libitum group (Kume et al., 2010). The amount of each amino acid in the DR plus either NEAAs or EAAs groups was supplemented to the level of the ad libitum group.

### 4.3 Blood and urine analysis

Blood glucose concentrations were measured using a Glutest sensor (Sanwa Kagaku, Nagoya, Japan). Urinary albumin excretion was measured using the Mikrofluoral Microalbumin Test (PROGEN, Heidelberg, Germany). Urinary creatinine was measured using the LabAssay Creatinine assay kit (Wako, Osaka, Japan). Plasma cystatin C levels were measured using the cystatin C (mouse) ELISA Kit (ALEXIS Biochemicals) (Kume et al., 2010). Glucose and insulin tolerance tests were performed as previously described (Tanaka et al., 2011).

### 4.4 Echocardiography

Ejection fraction was measured by transthoracic ultrasonography using a Vevo 2100 system (VisualSonics Inc).

### 4.5 Treadmill test

A treadmill running test was performed on a TMW‐4 mouse treadmill (MELQUEST) (Stockli et al., 2015). To evaluate exercise capacity, mice were subjected to running at 10 m/min for 10 min, followed by an increase in running speed of 1 m/min every 15 min. Total running time to exhaustion was determined.

### 4.6 Grip power measurement

Grip strength of both forelimbs was measured using a MK‐380CM/R grip strength meter (Muromachi Kikai Co. Ltd, Japan). Ten measurements were recorded, which were separated by 10‐min rest periods. The two highest and two lowest values were discarded from these measurements, and the remaining six values were averaged (Fukada et al., 2010).

### 4.7 Histological analyses

Fixed kidney samples embedded in paraffin were sectioned at 3μm thickness. Periodic acid–Schiff (PAS), hematoxylin–eosin (HE), Masson's trichrome, and immunohistochemical stains were performed as described previously (Kume et al., 2010). Antibodies to fibronectin (#AB2033, Chemicon, Temecula, CA), F4/80 (#MCA497GA, Serotec, Oxford, U.K.), CGL (#ab151769, Abcam, Cambridge, MA), CBS (#ab135626, Abcam, Cambridge, MA), klotho (Cosmo Bio. Co. Ltd., Tokyo, Japan). 8‐OHdG, and 4‐HNE (#MOG‐100P and #MHN‐100P, Japan Institute for the Control of Aging, Shizuoka, Japan) were used (Tanaka et al., 2011). Scanning electron microscopic analysis of glomeruli was performed using Hitachi S‐570 microscopes (Hitachi, Tokyo, Japan). Transmission electron microscopic analysis of skeletal muscle was performed with a Hitachi H‐7500 microscope (Hitachi, Tokyo, Japan). Histological scoring was performed by three independent nephrologists in a blinded manner.

### 4.8 Tissue amino acid profile

Frozen tissue samples were homogenized using a Precellys^®^ 24 homogenizer (Bertin Technologies, Montigny le bretonneux, France) in a cold 80% methanol solution containing L‐phenyl‐d5‐alanine as an internal standard and partitioned with chloroform to remove hydrophobic components such as lipids. The water‐soluble fractions were concentrated 10‐fold using a centrifugal evaporator and applied to the amino acid analysis. Amino acid concentrations in kidney homogenates were measured using high performance liquid chromatography‐electron spray ionization‐mass spectrometry (MS)‐MS (HPLC‐ESI‐MS/MS), followed by precolumn derivatization (Kume et al., 2014; Miyagi et al., 2011; Shimbo, Yahashi, Hirayama, Nakazawa, & Miyano, 2009).

### 4.9 Hydrogen sulfide measurements in kidney homogenates

Hydrogen sulfide (H_2_S) production capacity was measured using the lead sulfide method (Hine et al., 2015). Briefly, H_2_S production capacity was measured in 100 mg fresh tissue, in phosphate‐buffered saline supplemented with 10 mM Cys and 4 mM PLP. Lead acetate H_2_S detection paper (Sigma) was placed above the liquid phase in a closed microcentrifuge tube and incubated for 5 hr at 37°C.

### 4.10 RNA extraction and quantitative real‐time PCR

Quantitative real‐time PCR was performed as previously described (Tanaka et al., 2011). The levels of expression of each mRNA were normalized to the level of expression of glyceraldehyde 3‐phosphate dehydrogenase mRNA in the same sample. Primer sequences are listed in Supporting Information Table S5.

### 4.11 Statistical analyses

Differences among multiple data sets were analyzed by ANOVA, followed by Tukey's test. Pearson correlation coefficients were calculated to investigate associations among the indicated parameters. Analyses were performed using SPSS software, version 22. Kaplan–Meier analysis was carried out using GraphPad Prism version 6.00. All values are expressed as mean ± *SEM*. In all analyses, *p* < 0.05 was taken to indicate statistical significance.

## CONFLICT OF INTEREST

Yusuke Adachi and Kenji Nagao are employees of Ajinomoto Co., Inc.

## AUTHORS’ CONTRIBUTION

S.Y., K.Y., S.K., D.K., and S‐i.A. designed the study; S.Y., K.Y., M.Y.‐Y., Y.A., and K.N. carried out the experiments; S.Y. and S.K. analyzed the data and wrote the manuscript; N.T., M.C.‐K., N.O., and H.M. contributed to the study concept and the interpretation of the results; all authors contributed to discussion and revised the manuscript. All authors approve the final version of the manuscript.
