# Supplementary material for: Role of dietary amino acid balance in diet restriction‐mediated lifespan extension, renoprotection, and muscle weakness in aged mice
Source: Aging Cell. 2018 Jun 25;17(4):e12796. doi: 10.1111/acel.12796 (PMC6052467; doi:10.1111/acel.12796)
Supplement: Supplementary file 1 [file ACEL-17-na-s001.pdf]

Online Data Supplement

Role of dietary amino acid balance in diet restriction-mediated lifespan extension, renoprotection, and muscle weakness in aged mice

Running title: Dietary amino acid balance in kidney aging

Shohei Yoshida[1,a], Kosuke Yamahara[1,2,a], Shinji Kume[1], Daisuke Koya[3], Mako Yasuda-Yamahara[1,2], Naoko Takeda[1], Norihisa Osawa[1], Masami Chin-Kanasaki[1], Yusuke Adachi[4], Kenji Nagao[4], Hiroshi Maegawa[1], Shin-ichi Araki[1]

[1]Department of Medicine, Shiga University of Medical Science, Otsu, Shiga, Japan
[2]Department of Medicine IV, Faculty of Medicine, University of Freiburg, Freiburg, Germany
[3]Department of Diabetology & Endocrinology, Kanazawa Medical University, Kahoku-Gun, Ishikawa, Japan
[4]Frontier Research Labs, Institute for Innovation, Ajinomoto Co., Inc., Kawasaki, Kanagawa, Japan
[a]These authors contributed equally to this study.

Supplement Table 1. List of essential and non-essential amino acids

Essential amino acids	Non-essential amino acids
Histidine (His)	Alanine (Ala)
Isoleucine (Ile)	Arginine (Arg)
Leucine (Leu)	Aspartic acid (Asn)
Lysine (Lys)	Asparagine (Asp)
Methionine (Met)	Cysteine (Cys)
Phenylalanine (Phe)	Glutamine (Gln)
Threonine (Thr)	Glutamic acid (Glu)
Tryptophan (Trp)	Glycine (Gly)
Valine (Val)	Proline (Pro)
	Serine (Ser)
	Tyrosine (Tyr)

Supplement Table 2. Dietary composition of each dietary regimen

			Control	NEAA	EAA	EAA-Met
Casein			14.00 %	14.00 %	14.00 %	14.00 %
L-cystine			0.18 %	0.18 %	0.18 %	0.18 %
Corn starch			46.57 %	41.69 %	42.70 %	42.92 %
Alpha-corn starch			15.50 %	15.50 %	15.50 %	15.50 %
Glucose			10.00 %	10.00 %	10.00 %	10.00 %
Soy bean oil			4.00 %	4.00 %	4.00 %	4.00 %
Cellulose			5.00 %	5.00 %	5.00 %	5.00 %
Mineral mix			3.50 %	3.50 %	3.50 %	3.50 %
Vitamin mix			1.00 %	1.00 %	1.00 %	1.00 %
Choline bitartrate			0.25 %	0.25 %	0.25 %	0.25 %
Tert-Butylhydroquinone			0.0008 %	0.0008 %	0.0008 %	0.0008 %
Total			100.0 %	100.0 %	100.0 %	100.0 %
			Addition of dietary amino acids			
%, w/w	Amino acids in casein		Control	NEAA	EAA	EAA-Met
His	2.54	0.36 %	0.00 %	0.00 %	0.24 %	0.24 %
Ile	4.45	0.62 %	0.00 %	0.00 %	0.42 %	0.42 %
Leu	8.13	1.14 %	0.00 %	0.00 %	0.76 %	0.76 %
Lys・HCl	8.82	1.23 %	0.00 %	0.00 %	0.82 %	0.82 %
Met	2.43	0.34 %	0.00 %	0.00 %	0.23 %	0.00 %
Phe	4.50	0.63 %	0.00 %	0.00 %	0.42 %	0.42 %
Thr	3.81	0.53 %	0.00 %	0.00 %	0.36 %	0.36 %
Trp	1.08	0.15 %	0.00 %	0.00 %	0.10 %	0.10 %
Val	5.73	0.80 %	0.00 %	0.00 %	0.53 %	0.53 %
Ala	2.55	0.36 %	0.00 %	0.24 %	0.00 %	0.00 %
Arg	3.28	0.46 %	0.00 %	0.31 %	0.00 %	0.00 %
Asn・H2O	3.60	0.50 %	0.00 %	0.34 %	0.00 %	0.00 %
Asp	3.16	0.44 %	0.00 %	0.30 %	0.00 %	0.00 %
Cys-Cys	0.50	0.07 %	0.00 %	0.05 %	0.00 %	0.00 %
Gln	9.16	1.28 %	0.00 %	0.86 %	0.00 %	0.00 %
Glu	9.16	1.28 %	0.00 %	0.86 %	0.00 %	0.00 %
Gly	1.62	0.23 %	0.00 %	0.15 %	0.00 %	0.00 %
Pro	9.37	1.31 %	0.00 %	0.87 %	0.00 %	0.00 %
Ser	5.06	0.71 %	0.00 %	0.47 %	0.00 %	0.00 %
Tyr	4.85	0.68 %	0.00 %	0.45 %	0.00 %	0.00 %
AA Total	93.82	13.14 %	0.00 %	4.88 %	3.87 %	3.65 %
Starch	6.18	0.86 %				
Casein Total	100.0	14.0 %				

Supplement Table 3. Laboratory test data in each group of mice

	Young	Aged			
	Ad-libitum	Ad-libitum	Simple DR	NEAA	EAA
TP (g/dl)	4.76 ± 0.13	5.10 ± 0.12	4.06 ± 0.14*	4.86 ± 0.18	5.00 ± 0.23
Glob (g/dl)	1.26 ± 0.10	1.96 ± 0.29	1.10 ± 0.21	1.30 ± 0.19	1.10 ± 0.18
Ca (mg/dl)	9.36 ± 0.12	9.34 ± 0.11	9.00 ± 0.21	9.46 ± 0.27	9.82 ± 0.38
P (mg/dl)	9.20 ± 0.62	8.94 ± 0.80	9.96 ± 1.05	8.82 ± 0.46	9.30 ± 0.76
ALT (U/l)	25.6 ± 2.87	35.4 ± 5.80	50.0 ± 11.6	55.8 ± 12.5	43.2 ± 9.54
T-Bil (mg/dl)	0.26 ± 0.02	0.28 ± 0.02	0.28 ± 0.02	0.28 ± 0.02	0.32 ± 0.05
ALP (U/l)	42.0 ± 5.76	71.6 ± 12.3	63.0 ± 8.72	64.8 ± 8.45	90.0 ± 27.1
IGF-1	511.6 ± 26.0	890.4 ± 36.3*	452.0 ± 72.4	383.7 ± 68.3	474.0 ± 88.7

General serum test were measured with VetScan (ABAXIS). DR; diet restriction, NEAA; non-essential amino acid, EAA; essential amino acid, TP; total protein, Glob; globulin, ALT; Alanine aminotransferase, ALP; alkari phosphatase. Serum insulin-like growth factor-1 (IGF-1) was measured using an Elisa kit (#MG100, R&D Systems Minneapolis, MN). All data are expressed as mean ± SEM. *P < 0.05. vs the other groups. Differences among multiple data sets were analyzed by ANOVA followed by Tukey’s test.

Supplement Table 4. All-cause mortality and tumor prevalence at necropsy in the groups of aged mice

Groups	All-cause death	Tumor prevalence in all mice	Tumor prevalence in the dead mice
Ad-libitum (n=20)	12/20 (60.0%)	9/20 (45.0%)	9/12 (75.0%)
Simple DR (n=25)	5/25 (20.0%)	1/25 (4.0%)	1/5 (20.0%)
DR+NEAA (n=24)	4/24 (16.7%)	1/24 (4.2%)	1/4 (25.0%)
DR+EAA (n=30)	18/30 (60.0%)	2/30 (6.7%)	2/18 (11.1%)

DR; diet restriction, NEAA; non-essential amino acid, EAA; essential amino acid

Supplement Table 5. Primer sequences used in this study

Gene name	Forward	Reverse
Cystathionine γ-lyase (CGL)	5'-ttggatcgaaacacccacaaa-3'	5'-agccgactattgaggtcatca-3'
Cystathionine β-synthase (CBS)	5'-gggacaaggatcgagtctgga-3'	5'-agcactgtgtgataatgtggg-3'
Klotho	5'-aaccagccccttgaagggac-3'	5'-tgcacatcccacagatagac-3'
Glyceraldehyde 3-phosphate dehydrogenase (GAPDH)	5'-atggccttccgtgttcct-3'	5'-gcctgcttcaccaccttct-3'

Supplemental Figure 1

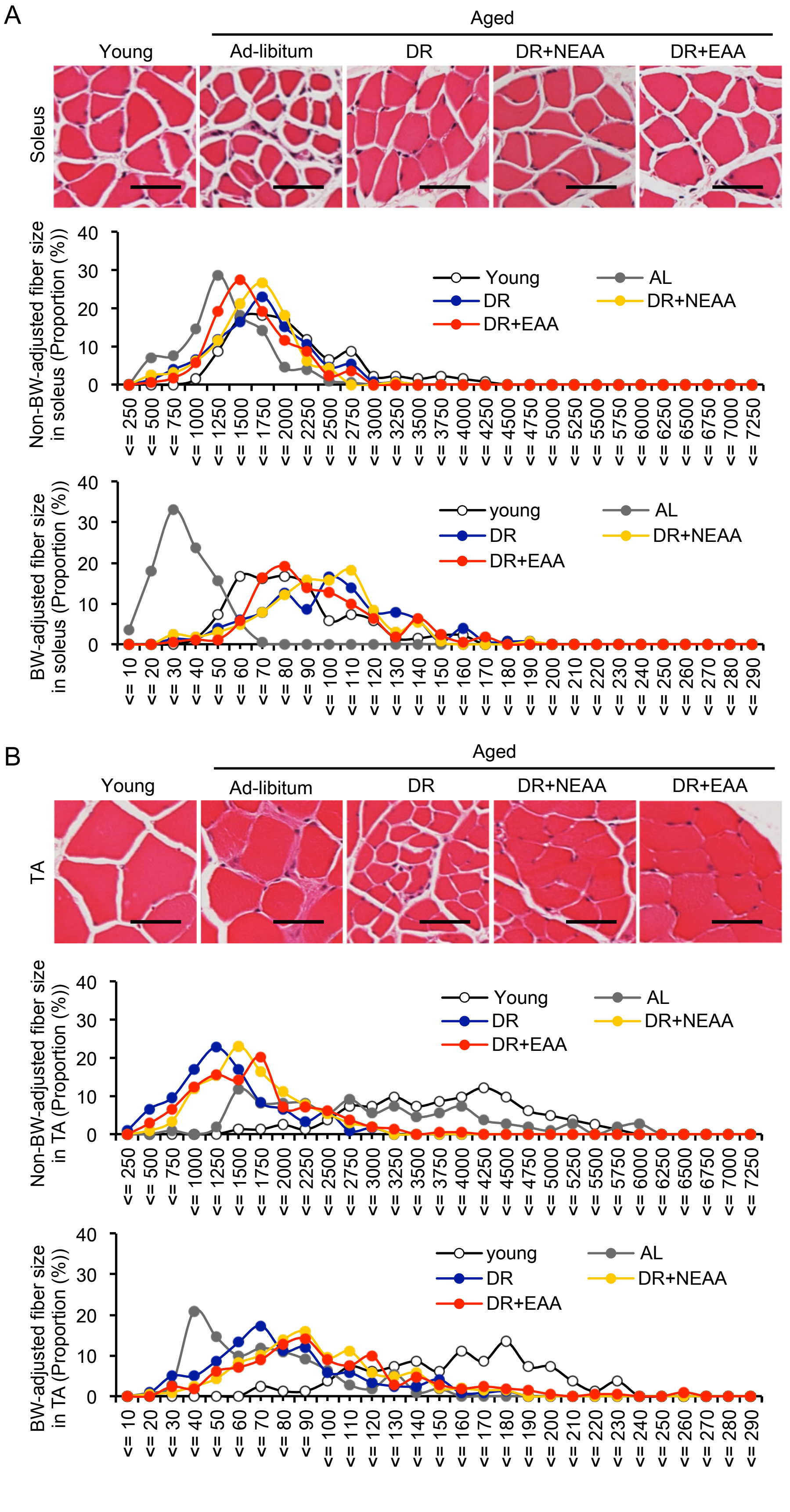

Supplemental Figure 1. Effects of dietary restriction and dietary amino acids on muscle fiber size in aged mice. Representative photomicrographs of hematoxylin-eosin-stained sections of soleus (A) and tibialis anterior (B) muscles in each group of mice. Cell size and count were measured using Image-Pro Plus 7.0 (Media Cybernetics, Bethesda, MD, USA). Data are shown as either body weight non-(BW)-adjusted or BW–adjusted fiber size. Error bar = 50 μm.

Supplemental Figure 2

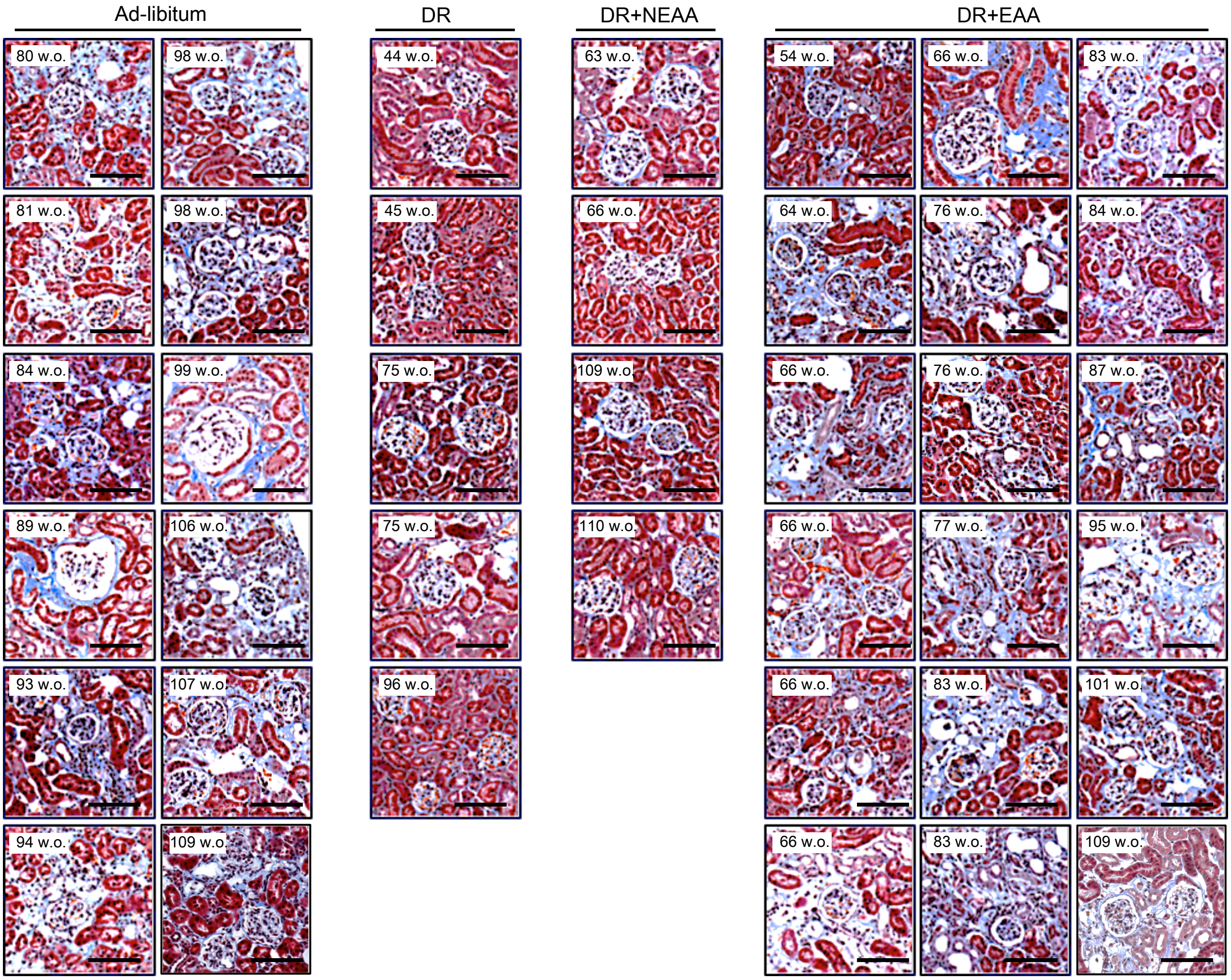

Supplemental Figure 2. Representative microphotographs of Masson's trichrome-stained kidney sections prepared following necropsy. The number in each picture indicates the age in weeks at death. Error bar = 100 μm.

Supplemental Figure 3

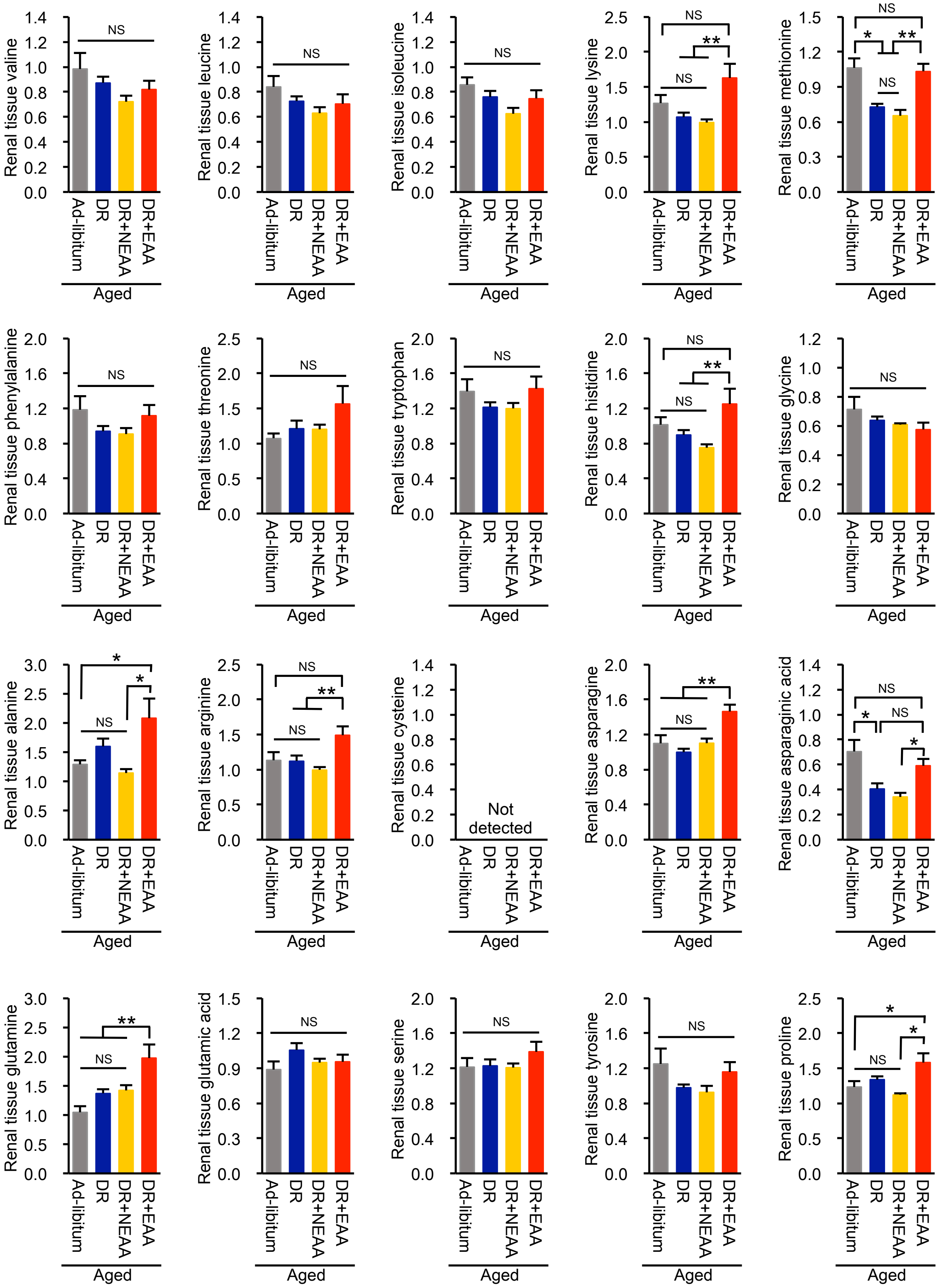

Supplemental Figure 3. Effects of DR and dietary amino acids on the renal amino acid profile in aged kidneys. Renal amino acid contents in the indicated mouse groups. All data are expressed as mean ± SEM. *P < 0.01 vs the indicated group. **P < 0.05 vs the indicated group. NS indicates statistically not significant.
